# High platelet-to-lymphocyte ratios in triple-negative breast cancer associates with immunosuppressive status of TILs

**DOI:** 10.1186/s13058-022-01563-7

**Published:** 2022-10-10

**Authors:** Hiroko Onagi, Yoshiya Horimoto, Asumi Sakaguchi, Daiki Ikarashi, Naotake Yanagisawa, Takayuki Nakayama, Tetsuya Nakatsura, Yumiko Ishizuka, Ritsuko Sasaki, Junichiro Watanabe, Mitsue Saito, Harumi Saeki, Takuo Hayashi, Atsushi Arakawa, Takashi Yao, Shigehisa Kitano

**Affiliations:** 1 Department of Human Pathology, Juntendo University School of Medicine, 2-1-1 Hongo, Bunkyo-ku, Tokyo, 113-0033 Japan; 2 Department of Breast Oncology, Juntendo University School of Medicine, Tokyo, Japan; 3 Division of Cancer Immunotherapy, Exploratory Oncology Research and Clinical Trial Center, National Cancer Center, 6-5-1 Kashiwanoha, Kashiwa, Chiba 277-8577 Japan; 4 Medical Technology Innovation Center, Juntendo University, Tokyo, Japan; 5 Division of Cancer Immunotherapy Development, Department of Advanced Medical Development, The Cancer Institute Hospital of Japanese Foundation for Cancer Research, 3-8-31 Ariake, Koto-ku, Tokyo, 135-8550 Japan

**Keywords:** Triple-negative breast cancer, Tumor-infiltrating lymphocyte, Regulatory T-cells, Platelet-to-lymphocyte ratio, Multiplexed fluorescent immunohistochemistry

## Abstract

**Background:**

Rating lymphocytes (TILs) are a prognostic marker in breast cancer and high TIL infiltration correlates with better patient outcomes. Meanwhile, parameters involving immune cells in peripheral blood have also been established as prognostic markers. High platelet-to-lymphocyte ratios (PLRs) and neutrophil-to-lymphocyte ratios (NLRs) are related to poor outcomes in breast cancer, but their mechanisms remain unknown. To date, TILs and these parameters have been examined separately.

**Methods:**

We investigated the relationship between TILs and the peripheral blood markers, PLR and NLR, in the same patients, using surgical specimens from 502 patients with invasive breast carcinoma without preoperative chemotherapy. For analysis of triple-negative breast cancer (TNBC) patient outcomes, 59 patients who received preoperative chemotherapy were also examined. For immune cell profiling, multiplexed fluorescent immunohistochemistry (mfIHC) of CD3, CD4, CD8, FOXP3 and T-bet, was conducted.

**Results:**

A positive correlation between PLR and TIL was observed in TNBC (*P* = 0.013). On mfIHC, tumors in patients with high PLR and NLR contained more CD3^+^CD4^+^FOXP3^+^ T-cells (*P* = 0.049 and 0.019, respectively), while no trend was observed in CD8^+^ T-cells. TNBC patients had different patterns of outcomes according to TIL and PLR, with the TIL-high/PLR-low group having the lowest rate of disease relapse and death, and the longest distant metastasis-free and overall survivals, while the TIL-low/PLR-high group had the shortest survivals.

**Conclusions:**

Our data suggest that the combination of PLR with TIL assessment may enable more accurate prediction of patient outcomes with TNBC.

**Supplementary Information:**

The online version contains supplementary material available at 10.1186/s13058-022-01563-7.

## Background

Tumor-infiltrating lymphocytes (TILs) are an established prognostic marker, particularly in patients with triple-negative breast cancer (TNBC). Reflecting the host immune response to the tumor, patients with tumors with a high TIL infiltration tend to have better outcomes [1, 2]. Meanwhile, immune checkpoint inhibitors (ICIs) have recently been introduced for TNBC with PD-L1^+^ TILs in combination with chemotherapeutic agents [3, 4]. A critical role of TILs in local tumor immunity is suggested as TIL levels are a predictive factor for ICI treatments [5], although factors attracting TILs are still largely unknown despite numerous studies [6, 7]. While the importance of TILs has been well recognized, there are some obstacles in their assessment, especially for patients with recurrent breast cancer. TILs are usually evaluated in the primary tumor, which may have been taken several years prior, since biopsies from recurrent lesions can be difficult to obtain. Additionally, even if a biopsy from a recurrent site is evaluated, it is still unclear whether it can be interpreted in the same way as the primary lesion because the degree of TIL infiltration and the profile of immune cells might differ in relation to the metastatic organ [8]. Therefore, establishing simple methods to evaluate the real time anti-tumor immune response in patients with recurrent disease, such as liquid biopsies in the form of peripheral blood sampling, is eagerly needed.

Parameters involving immune cells in the peripheral blood have been studied across various cancers in relation to patient outcomes. A number of studies have shown that a high neutrophil-to-lymphocyte ratio (NLR) is associated with poor outcomes with breast cancer and other cancers [9–11]. The platelet-to-lymphocyte ratio (PLR) has also been studied, and for breast cancer patients, both NLR and PLR are now established prognostic markers across all intrinsic subtypes [10, 12, 13]. In relation to therapeutic effect, a high NLR reportedly correlates with poor responsiveness to systemic treatments in breast cancer patients [11, 14] and we also previously reported a similar trend in eribulin-based and CDK4/6 inhibitor-based treatments [15, 16]. Lymphocyte activity is also recognized as crucial and high lymphocyte counts are associated with better response to chemotherapies in patients with metastatic breast cancer [17–19]. As for the mechanism of action, NLR and PLR indicate a degree of inflammation, which might exert a negative impact on the host immune system. Neutrophils may also suppress the activities of lymphocytes and consequently promote tumor progression [20, 21]. In vivo data for PLRs have suggested that platelet activation might suppress the functions of natural killer cells [22]. Moreover, other studies indicate platelets promote tumor proliferation, invasion and angiogenic signaling [23, 24]. Nevertheless, the mechanism underlying the association between immune cell parameters in peripheral blood and patient outcomes or treatment effects remains largely unknown.

As described above, TILs and peripheral blood parameters such as NLR and PLR, have been shown to be prognostic factors. However, to date they have been largely examined separately with only a few studies investigating the relationships between them in the same individual. Employing gene expression profiling, one report found a negative correlation between the cytolytic activity of TIL and PLR [25]. Another study reported that CD8^+^ T-cell counts in TILs correlated with the absolute lymphocyte count in peripheral blood [26]. Although these reports suggest a possible association between the local tumor immune status and systemic immune cell counts, investigation of this issue is still insufficient and requires clarity.

Therefore, in the present study, we investigated the relationships between TILs and peripheral blood markers using a large number of surgical specimens. Considering the prognostic properties of TILs and PLR/NLR, we initially predicted a negative correlation between them. However, contrary to our hypothesis, we found a positive correlation between TIL and PLR among subtypes of TNBC, and so we further conducted multiplexed fluorescent IHC (mfIHC) for immune cell profiling.

## Methods

### Patients

In total, 502 patients with invasive breast carcinoma, pathologically larger than 5 mm in diameter, who underwent curative surgery without preoperative chemotherapy at Juntendo University Hospital from 2012 through 2018 were investigated. Subtypes among the 502 patients were as follows: 300 patients were luminal human epidermal growth factor receptor 2 (HER2)-negative (Lum); 28 were luminal HER2-positive (LumH); 57 were HER2 type (HER); and 117 were triple-negative (TN). The clinicopathological features of these patients are shown in Additional file 1. All patients were Asian women. This study was carried out with approval from the ethics committee of Juntendo University Hospital (no: 19-182). Patients could see the research plan on the hospital website and were offered the choice to opt out of the study at any time.

### Pathological assessment and IHC

Pathological examinations were carried out by two pathologists at our hospital. Tumor grade was judged based on the Nottingham Histologic Score system. A tumor was considered positive for estrogen receptor or progesterone receptor if it had 1% or more staining of the cancer cell nuclei. HER2 was considered positive if the entire cell membrane of more than 10% of tumor cells showed strong staining, or *HER2/neu* gene amplification was confirmed by fluorescence in situ hybridization. In the current study, all tumors were categorized into Lum, LumH, HER and TN groups, based on the results of estrogen receptor, progesterone receptor and HER2 evaluation. The Ki67 labelling index was assessed from a hotspot within a high-powered field.

TIL levels were determined using hematoxylin and eosin-stained tumor surgical specimens, based on recommendations made by an International TILs Working Group [27]. Briefly, TILs in the stromal compartment (% stromal TILs), using the area of stromal tissue as a denominator, were determined semi-quantitatively. TILs were examined within the borders of the invasive tumor, and the average TIL numbers in the tumor area, not focusing on hotspots, were assessed. For PD-L1 detection, an anti-PD-L1 monoclonal antibody (clone SP142; Abcam, Tokyo, Japan) was used for IHC. Membrane staining of stromal immune cells was semi-quantitative. Scoring of PD-L1 was assessed as: 0, < 1%; 1, ≥ 1 to < 5%; 2, ≥ 5 to < 10%; and 3, ≥ 10%, based on criteria applied clinically for breast cancer [3], i.e., IC0–IC3.

### MfIHC and image analysis

For immune cell profiling we conducted mfIHC. Details of the procedure have been described previously [28]. Tyramide signal amplification was used employing an Opal IHC kit (PerkinElmer, Waltham, MA) according to the manufacturer's instructions. We previously constructed a T-cell panel, comprising CD3, CD4, CD8, FOXP3 and T-bet. This panel also included cytokeratin to differentiate tumor and stromal areas, and DAPI for staining nuclei [28]. Primary antibodies used were: CD3 (clone SP7, Abcam, Tokyo, Japan), CD4 (clone 4B12, Leica Microsystems, Tokyo, Japan), CD8 (clone 4B11, Leica Microsystems), FOXP3 (D6O8R, Cell Signaling Technology, Danvers, MA), T-bet (clone 4B10, Santa Cruz Biotechnology, Dallas, CA) and cytokeratin (clone AE1/AE3, Dako Agilent Technologies, Santa Clara, CA). Employing an automated imaging system (Vectra ver. 3.0, PerkinElmer), an average of 20 areas at × 200 were captured for each patient. An image analyzing software program (InForm, PerkinElmer) segmented cancer tissue into cancer cell nests (intratumoral) and the framework (stromal) region, and detected immune cells with specific phenotypes. Representative images of tissue segmentation and cell phenotype recognition are shown in Additional file 2. Infiltrating immune cells were quantified using an analytic software program (Spotfire, TIBCO, Palo Alto, CA) and then calculated per area.

### Evaluation of immune cells in peripheral blood

Peripheral blood samples were obtained before treatments and the number of lymphocytes, PLR, and NLR were calculated from the laboratory data. Hematological analysis according to the flow cytometry method for measuring and differentiating cell types in whole blood was conducted using XE-5000 (Sysmex Corporation, Japan).

### Statistical analysis

SAS version 9.4 and JMP 14.2 statistical software (SAS Institute, Inc., Cary, NC) were used for statistical analyses. The associations between PLR, NLR and TIL were examined in all patients and in each of the four subgroups (Lum, LumH, HER, TN), employing correlation coefficients and linear regressions. PLR, NLR and TIL were log-transformed and Pearson's correlation coefficient was calculated. No adjustment factors were taken into account in the linear regression. Patients with a TIL of 0% were excluded from the analysis because they could not be log-transformed. For a test of independence among groups, the Pearson’s chi-square test was employed. A logistic regression model was constructed to identify factors characterizing patients who developed distant metastases. For the full-model analysis, we first selected variables according to their clinical significance: age, tumor size, lymph node metastasis, tumor grade, TIL, PLR and administration of adjuvant chemotherapy. For patient outcomes, Kaplan–Meier curves were estimated and the Wilcoxon’s test was applied for comparisons of survival distributions between patient groups. A *P* < 0.05 was considered statistically significant.

## Results

### Comparisons of TIL, PLR and NLR among subtypes

In all 502 patients, the mean values of TIL, PLR and NLR were 31% (range, 0–100%), 153 (46–477), and 2.4 (0.7–11.9), respectively. Additional file 3 shows comparisons of these parameters among subtypes. The Lum group had significantly less TILs than the other subtypes (*P* < 0.001), while there were no differences among the remaining three groups (mean TIL values in Lum, LumH, HER, and TN were 22%, 46%, 48%, and 43%, respectively). In contrast, there were no differences in PLR or NLR among the subtypes (PLR: 153, 164, 146, and 153; NLR: 2.2, 2.3, 2.4, and 2.5, respectively).

### Relationships between TILs and PLR/NLR

**Fig. 1:**
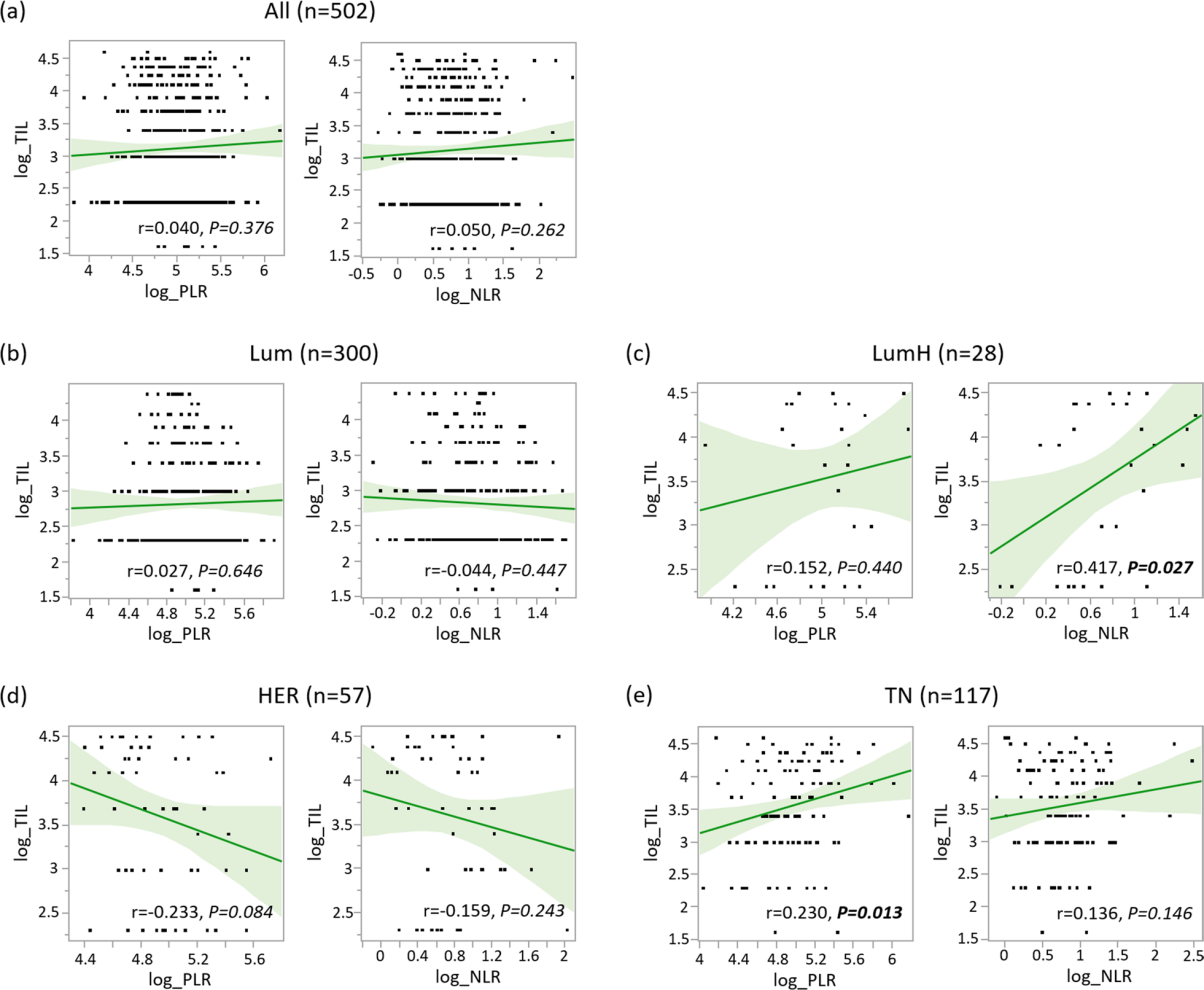
Relationships between tumor-infiltrating lymphocytes and platelet-to-lymphocyte ratio, and TILs and neutrophil-to-lymphocyte ratio. Relationships between tumor-infiltrating lymphocytes (TILs) and platelet-to-lymphocyte ratio (PLR), and TILs and neutrophil-to-lymphocyte ratio (NLR) (all log-transformed) are shown in (**a**) all patients, (**b**) luminal HER2-negative (Lum), (**c**) luminal HER2-positive (LumH), (**d**) human epidermal growth factor receptor 2 type (HER), and (**e**) triple-negative (TN) tumors. Green lines and light green areas indicate regression lines and 95% confidence intervals, respectively. “*r*” indicates the Pearson's correlation coefficient

Next, we examined the relationships between TIL and PLR, and TIL and NLR (Fig. 1). There was no trend when compared in all patients (*n* = 502), however, when divided according to subtype, a significant positive correlation between PLR and TIL was observed in TN tumors (*P* = 0.013). As a similar trend was confirmed for NLR and TIL in TN tumors, we determined to conduct further analysis of TN tumors, employing multiplex staining. Meanwhile, a significant correlation between TIL and NLR was also observed in LumH tumors (*P* = 0.027). However, due to its low frequency, we could not obtain a sufficient number of samples for further investigation.

### Immune cell profiles in TN in relation to PLR and NLR

**Fig. 2:**
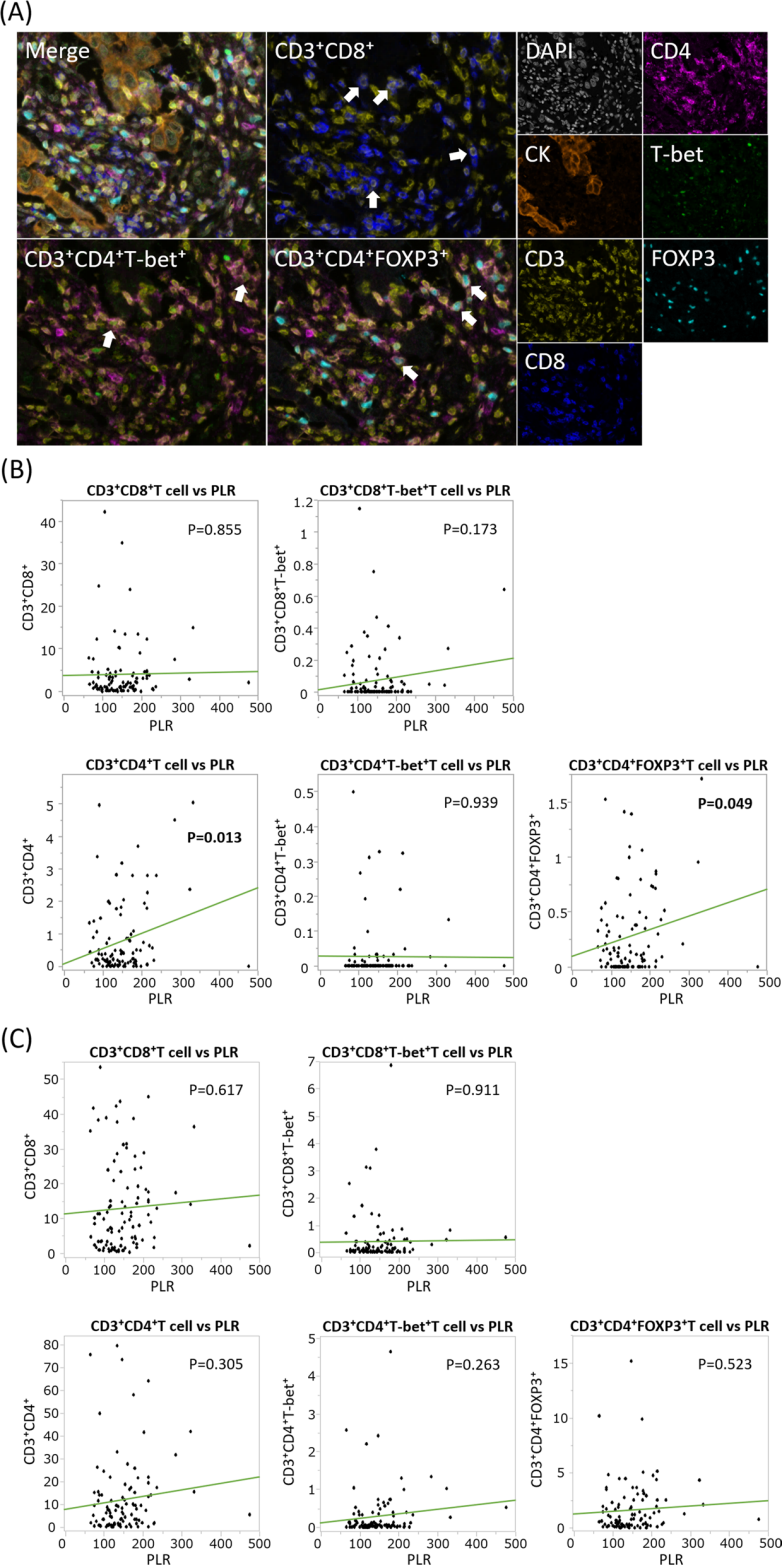
Relationships of T-cell lymphocyte infiltration and platelet-to-lymphocyte ratio. **a** Representative images of CD3^+^CD8^+^, CD3^+^CD4^+^T-bet^+^, and CD3^+^CD4^+^FOXP3^+^ T-cell detections. **b** and **c** Relationships between a variety of T-cell lymphocyte infiltrations and platelet-to-lymphocyte ratio (PLR). Assessments in (**b**) cancer and (**c**) stromal areas are separately indicated. White arrows indicate respective corresponding immune cells. Green lines indicate regression lines

Among 117 TN tumors, 109 samples available for mfIHC were investigated. As a result, data from 99 patients were available for assessment. Representative images of mfIHC and results of this analysis are shown in Fig. 2. While there was no trend in the relationships between PLR and CD3^+^CD8^+^ T-cells, CD3^+^CD4^+^ T-lymphocyte infiltration in the cancer area was positively correlated with PLR (*P* = 0.013). Among CD4^+^ T-cells, CD3^+^CD4^+^FOXP3^+^ T-cells retained this correlation with PLR (*P* = 0.049), while the trend diminished in CD3^+^CD4^+^T-bet^+^ T-cells (*P* = 0.939). These findings were observed only in the cancer area and not in the stromal area. For NLR, similar trends were observed, as both CD3^+^CD4^+^ and CD3^+^CD4^+^FOXP3^+^ T-cell infiltration had a positive correlation with NLR (*P* = 0.037 and 0.019, respectively) (Additional file 4).

### Relationships between PD-L1 expression and PLR/NLR

Next, the relationships between PD-L1 protein expression in immune cells and both PLR and NLR were examined. PD-L1 was assessed by conventional IHC, since the number of channels for mfIHC was limited. As shown in Additional file 5, positive correlation trends between PD-L1 expression and PLR/NLR were observed, although these relationships were not statistically significant (*P* = 0.067 and 0.069, respectively.).

### Comparison of TIL and PLR/NLR values according to development of metastatic disease

Among the 117 TN patients, 10 patients (8.5%) developed distant metastasis during the mean observation period of 49 months (range, 1–114). Seven patients (6.0%) died due to breast cancer. Additional file 6 shows the relationships of TIL, PLR, and NLR with the occurrence of metastases. TIL, PLR, and NLR in patients with distant metastases versus those free from distant metastases were: TIL, 37% versus 44%; PLR, 175 versus 151; and NLR, 2.8 versus 2.5, respectively. Patients with distant metastases had lower TIL involvement and higher PLRs and NLRs, although there were no significant differences.

### Combination of TIL and PLR as a prognostic marker for TNBC patients

Finally, we attempted to evaluate a combination of TIL and PLR as a potential prognostic marker for patients with TNBC. For this purpose, we also employed patients with TNBC who received preoperative chemotherapy (*n* = 59) and underwent curative surgery during the same period, since the sample size, including cases with distant metastases, was too small in the aforementioned analyses. Clinicopathological features of the 176 patients are shown in Table 1. For patients receiving preoperative chemotherapy, TIL was evaluated in biopsy specimens taken before the systemic treatments. During the mean observation period of 49 months (range, 1–114), 29 patients developed distant metastases (16.5%) and 23 patients (13.1%) died due to breast cancer. Patients with large tumor size, lymph node metastasis, low TIL, or administration of adjuvant chemotherapy had a significantly higher frequency of development of distant metastasis (*P* < 0.001, *P* < 0.001, *P* = 0.011 and *P* = 0.032, respectively; Table 1). Among patients who received adjuvant chemotherapy, a full regimen (anthracycline followed by taxane) was also a factor (*P* = 0.041). On multivariate analysis, tumor size, lymph node metastasis and TIL remained as independent factors associated with development of metastatic disease (*P* < 0.001, *P* = 0.014 and *P* = 0.012, respectively).

**Table 1:** Clinicopathological features of the 176 patients with triple-negative breast cancer

Variables	All patients	Met	Non-met	Univariate			Multivariate		
				OR	95% CI	*P *value	OR	95% CI	*P *value
n	176	29	147						
Age, mean (range), y	58.9 (24–89)	55.3	59.6	0.3^b^	0.04–1.5	0.140	0.3^b^	0.02–4.8	0.400
Histology									
NST	141 (80%)	23	118	0.9	0.4–2.7	0.906			
Others	35 (20%)	6	29						
Tumor size, mean (range), mm	21.9 (0–75)	34.2	19.5	34.4^b^	6.2–214.7	< 0.001	60.6^b^	5.5–909.1	< 0.001
Lymph node metastasis									
Yes	52 (30%)	17	35	4.4	1.9–10.3	< 0.001	3.7	1.3–10.9	0.014
No	121 (70%)	12	109						
Not evaluated	3	0	3						
Tumor grade									
High	70 (48%)	16	54	1.9	0.8–4.9	0.151	1.6	0.5–5.0	0.453
Low	67 (52%)	9	58						
Not evaluated	39	4	35						
Ki67 LI, mean (range), %	57.3 (0–100)	61.7	56.4	1.9^b^	0.5–8.8	0.376			
TIL, mean (range), %	43.0 (0–100)	31.6	45.2	0.1	0.02–0.6	0.011	0.1	0.004–0.6	0.012
ALC, median (range), μL^−1^	1640 (519–3917)	1589	1645	0.4^b^	0.03–3.9	0.460			
PLR, median (range)	143 (56–477)	145	137	4.3^b^	0.3–47.2	0.241	3.9^b^	0.1–102.0	0.435
NLR, median (range)	2.1 (0.9–11.9)	2.2	2.1	1.8^b^	0.1–29.8	0.702			
Adjuvant chemotherapy									
Yes	132^a^ (75%)	26	106	3.4	1.1–14.6	0.032	2.3	0.5–13.5	0.272
No	44 (25%)	3	41						
Chemotherapy regimens^a^									
A + T	79 (60%)	20	59	2.7^c^	1.0–7.7	0.041			
A alone	47 (36%)	5	42						
T alone	6 (4%)	1	5						

Values are *n* (%) unless otherwise indicated

^a^Includes 59 patients who received preoperative chemotherapy

^b^Range of the odds ratio

^c^A + T versus A alone/T alone

*A* anthracycline-based regimen, *ALC* absolute lymphocyte count, *CI* confidence interval, *LI* labeling index, *Met* patients who developed distant metastases, *NLR* neutrophil-to-lymphocyte ratio, *non-Met* patients free from distant metastases, *NST* no special type, *OR* odds ratio, *PLR* platelet-to-lymphocyte ratio, *T* taxane, *TIL* tumor-infiltrating lymphocyte

Although PLR was not a statistically significant factor, the combination of TIL and PLR was further assessed due to the above reasons. Table 2 shows the distribution of patients with and without the development of distant metastases and breast cancer-related deaths according to TIL and PLR. TNBC patients (*n* = 176) were divided into four groups, according to median TIL and PLR values: Group 1, TIL-high/PLR-low (*n* = 31); Group 2, TIL-high/PLR-high (*n* = 43); Group 3, TIL-low/PLR-low (*n* = 60); and Group 4, TIL-low/PLR-high (*n* = 42). There was a significant difference in the distribution of recurrence rates among the four groups (*P* = 0.026), with the rate being lowest in Group 1 (3.2%) and highest in Group 4 (28.6%). When two groups were compared with each other, significant differences were observed between Group 1 and 3 (*P* = 0.044) and Group 1 and 4 (*P* = 0.005). Similarly, mortality rates differed among the groups (*P* = 0.022); Group 4 had a significantly higher death rate than Group 1 and 2 (*P* = 0.009 and 0.041, respectively).

**Table 2:** Distribution of the development of distant metastases and breast cancer-related deaths according to tumor-infiltrating lymphocytes and platelet-to-lymphocyte ratio

	*n*	Met	*P* value	Death	*P* value
Group 1 (TIL-high/PLR-low)	31	1 (3.2%)		1 (3.2%)	
Group 2 (TIL-high/PLR-high)	43	5 (11.6%)	0.026^a^	4 (9.3%)	0.022^a^
Group 3 (TIL-low/PLR-low)	60	11 (18.3%)		7 (11.7%)	
Group 4 (TIL-low/PLR-high)	42	12 (28.6%)		11 (26.2%)	

^a^Comparisons among the four group﻿s

*Met* patients who developed distant metastases, *PLR* platelet-to-lymphocyte ratio, *TIL* tumor-infiltrating lymphocytes

**Fig. 3:**
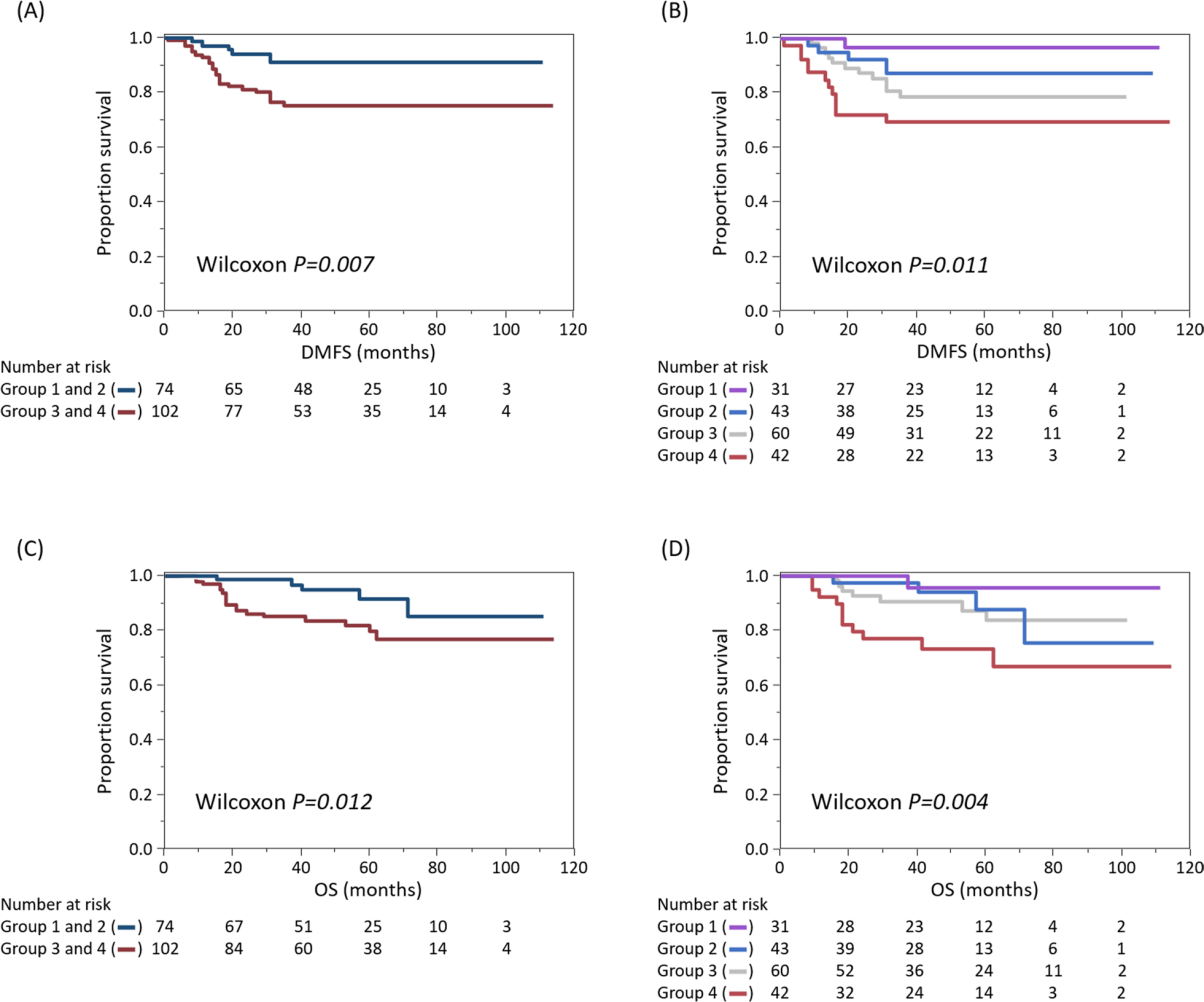
Kaplan–Meier curves of triple-negative breast cancer patients (*n* = 176) according to tumor-infiltrating lymphocytes (TILs) and platelet-to-lymphocyte ratios (PLRs). Patient outcomes according to patient groups, based on TIL and PLR are shown. Group 1, TIL-high/PLR-low (*n* = 31); Group 2, TIL-high/PLR-high (*n* = 43); Group 3, TIL-low/PLR-low (*n* = 60); and Group 4, TIL-low/PLR-high (*n* = 42). **a** Distant metastasis-free-survival (DMFS) and, **c** overall survival (OS) according to TILs. Dark blue and red lines indicate TIL-high patients (Groups 1 and 2), and TIL-low patients (Groups 3 and 4), respectively. **b** Comparison of DMFS and, **d** OS, according to the four patient groups

Finally, we drew Kaplan–Meier curves for the four groups (Fig. 3). When TIL-high (Groups 1 and 2) and -low (Groups 3 and 4) patients were compared, the TIL-high population had longer survival both for distant metastasis-free survival (DMFS) and overall survival (OS) (*P* = 0.007 and 0.012, respectively). Meanwhile, when divided into four groups according to TILs and PLR, there were also significant differences among the groups in DMFS and OS (*P* = 0.011 and 0.004, respectively), with Group 1 and Group 4 having the longest and the shortest survival, respectively.

## Discussion

In the current study, we found a positive correlation between TIL and PLR in TNBC patients. TILs in patients with high PLR predominantly contained CD3^+^CD4^+^FOXP3^+^ T-cells, i.e., regulatory T-cells (Tregs). Moreover, PD-L1 expression tended to be high in this population. These data suggest the possibility that local tumor-immunity may be suppressed in TNBC patients with high PLR. To the best of our knowledge, this is the first study to directly demonstrate such a detailed relationship between TILs and peripheral blood markers, employing mfIHC. Previously, Romero-Cordoba et al. [25] performed gene expression profiling of 54 TNBC tumors and proposed that TNBC can be classified into three immuno-clusters. They found that PLR correlated negatively with TIL cytolytic activity and tumor inflammation signature. However, their results are not necessarily contradictory to ours, since they presumably observed the results of suppressed tumor-immunity at primary tumors in patients with high PLR. Nevertheless, immune features in their study were indirectly defined and assessed by clustering of genetic analysis. In contrast, employing a larger number of patients, we revealed that Tregs, as strictly defined by mfIHC, were highly infiltrative. Hence, we believe that our results will give useful information to other researchers and clinicians when planning further studies. Moreover, our data suggest that the combination of PLR data with conventional TIL evaluation may enable more accurate prediction of patient outcomes for TNBC patients. In other words, while high TIL infiltration is widely considered to be a favorable prognostic factor, there may be a subset of patients with high PLR among TIL-high TNBC patients, who has a poor outcome [1, 2].

It is noteworthy that PLR and NLR did not reflect the degree of CD8^+^ T-cell infiltration. Instead, Tregs were dominant in PLR/NLR-high TNBC. Platelets reportedly suppress NK-cell function, including cytotoxicity and IFN-γ production, by their surface proteins and secreted cytokines such as TGFβ [29–31]. Moreover, platelets have been reported to activate Tregs via platelet factor 4, a platelet-derived CXC chemokine [32]. In addition, platelets also interact with tumor cells. Tumor cells activate platelets directly or through cytokine secretion, and conversely, platelets secrete factors, such as fibronectin, that promote tumor invasion and metastasis [23]. Similarly, neutrophils also promote Treg differentiation [33] and induce apoptosis of CD8-positive T-cells [34, 35]. Therefore, considering these effects of platelets and neutrophils, the local immune response is presumably suppressed in patients with high PLR and NLR. However, to what extent PLR and NLR values, as assessed by peripheral blood, reflect the effects of platelets and neutrophils in local lesions remains unknown. Nevertheless, our data indicate that the quality of TILs, reflecting local tumor-immunity, might be assessed, to some extent, by peripheral blood markers, such as PLR, without mfIHC.

It is also interesting that negative correlations of TIL with PLR and NLR were observed in HER, unlike TN, although there was no statistical difference. The role of inflammation in tumor immunity may be different between TN and HER subtypes and further studies should be conducted with mfIHC, as we did, to clarify the detailed profiling of TILs in HER. In addition, the role of PLR/NLR as a prognostic marker may need to be considered separately between TN and HER subtypes.

There are some limitations in the current study. We could not conduct a detailed profiling of the immune cells in peripheral blood, such as classification of lymphocytes by flow cytometry. In the evaluation of TIL for patients who received pre-operative chemotherapy, biopsy specimens were assessed but it may be necessary to consider some discrepancies between biopsy and surgical specimens. To establish the combination of TIL and PLR as a prognostic marker for TNBC patients, further studies with larger sample sizes are needed to validate our results and determine cut-off values.

## Conclusions

TNBC patients with high PLR, whose tumors contained more Treg, had worse outcomes. Our data indicate that PLR ought to be assessed in combination with conventional TIL evaluation for prediction of patient outcomes.

## Supplementary Information

**Additional file1**. Clinicopathological features of the 502 patients.

**Additional file2**. Representative images of tissue segmentation and cell phenotype recognition. (a) Employing an image analyzing software program, tissue segmentation was conducted. Tumor area and stromal area were recognized with red and green, respectively. (b) Cell phenotyping was also conducted with markers specific to each cell; cancer cells (orange), stromal cells (green), CD4+ T-cells (yellow), CD8+ T-cells (pink). A combination of the tissue segmentation and cell phenotyping enabled separate assessments of infiltrating immune cells in intratumoral and stromal regions.

**Additional file3**. Comparisons of tumor-infiltrating lymphocytes and parameters in peripheral blood according to subtype. Comparisons of tumor-infiltrating lymphocytes (TILs), platelet-to-lymphocyte ratios (PLRs), and neutrophil-to-lymphocyte ratios (NLRs) according to subtypes are shown (luminal HER2-negative [Lum], n = 300; luminal HER2-positive [LumH], n = 28; human epidermal growth factor receptor 2 type [HER], n = 57; triple-negative [TN], n = 117). Horizontal bars indicate mean values. Mean TIL, PLR and NLR values in Lum, LumH, HER, and TN were 22%, 46%, 48%, and 43%; 153, 164, 146, and 153; and 2.2, 2.3, 2.4, and 2.5, respectively.

**Additional file4**. Relationships between T-cell lymphocyte infiltration and neutrophil-to-lymphocyte ratio. Description of data: Relationships between a variety of T-cell lymphocyte infiltration and neutrophil-to-lymphocyte ratio (NLR) in (a) cancer and (b) stromal areas are shown. Green lines indicate regression lines.

**Additional file5**. Relationships between PD-L1 protein expression in immune cells and both PLR and NLR. Relationships between PD-L1 protein expression in immune cells (IC) and both platelet-to-lymphocyte ratio (PLR) and neutrophil-to-lymphocyte ratio (NLR). Blue lines and light blue areas indicate regression lines and 95% confidence intervals, respectively. “r” indicates the Pearson's correlation coefficient.

**Additional file6**. Comparisons of tumor-infiltrating lymphocytes, platelet-to-lymphocyte ratio and neutrophil-to-lymphocyte ratio according to development of metastatic disease. Tumor-infiltrating lymphocytes (TIL), platelet-to-lymphocyte ratio (PLR) and neutrophil-to-lymphocyte ratio (NLR) were compared between patients free from distant metastases (non-Met; n = 107) and patients who developed distant metastases (Met; n = 10).

## Data Availability

Data sharing is not applicable to this article as no datasets were generated.

## Abbreviations

DMFS: Distant metastasis-free survival
HER: HER2 type
HER2: Human epidermal growth factor receptor 2
ICIs: Immune checkpoint inhibitors
IHC: Immunohistochemistry
Lum: Luminal HER2-negative
LumH: Luminal HER2-positive
mfIHC: Multiplexed fluorescent immunohistochemistry
NLR: Neutrophil-to-lymphocyte ratio
OS: Overall survival
PLR: Platelet-to-lymphocyte ratios
TILs: Tumor-infiltrating lymphocytes
Tregs: Regulatory T-cells
TN: Triple-negative
TNBC: Triple-negative breast cancer
