# Supplementary figures and images for: High platelet-to-lymphocyte ratios in triple-negative breast cancer associates with immunosuppressive status of TILs
Source: Breast Cancer Res. 2022 Oct 10;24:67. doi: 10.1186/s13058-022-01563-7 (PMC9552414; doi:10.1186/s13058-022-01563-7)

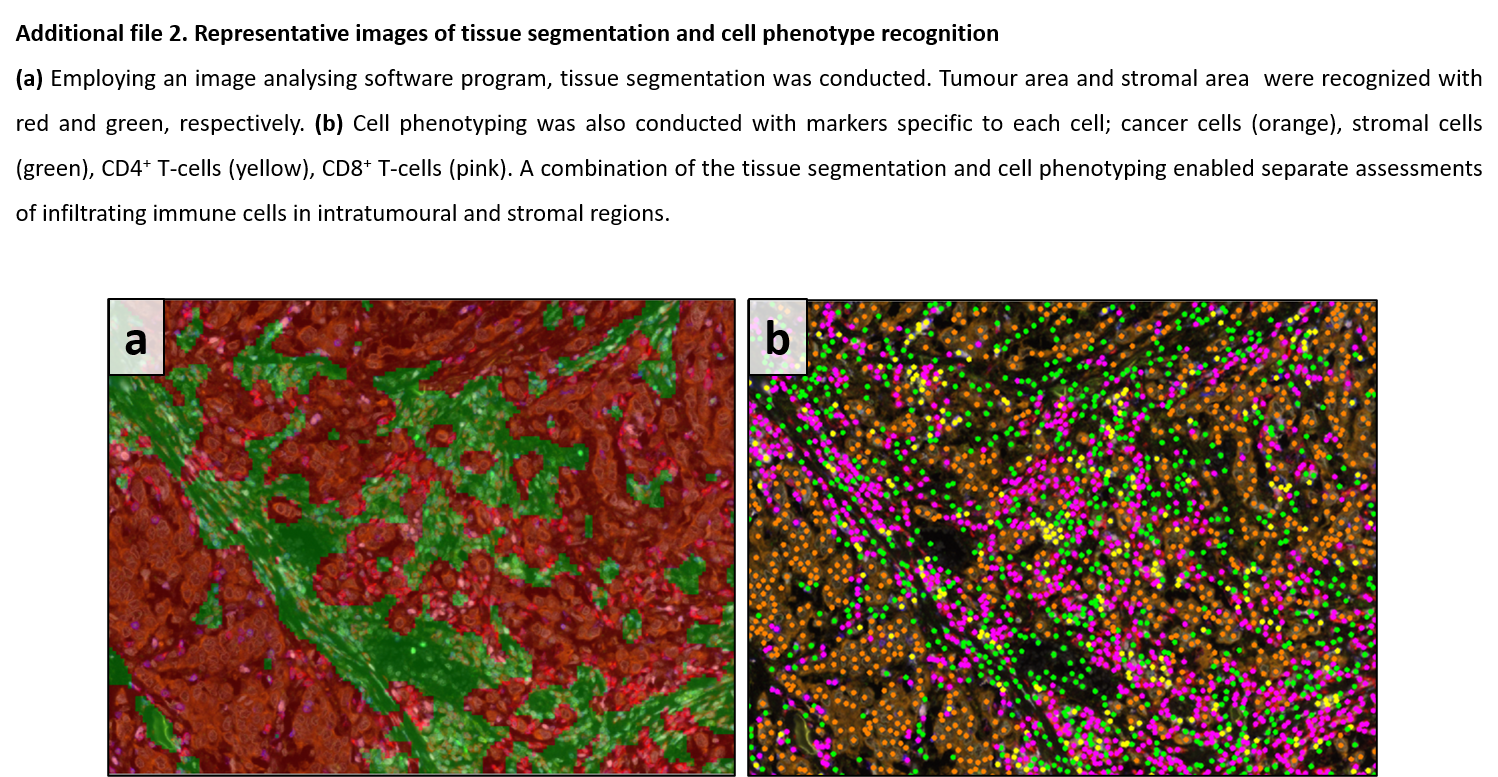

Supplement: Supplementary file 2 — Additional file2. Representative images of tissue segmentation and cell phenotype recognition. (a) Employing an image analyzing software program, tissue segmentation was conducted. Tumor area and stromal area were recognized with red and green, respectively. (b) Cell phenotyping was also conducted with markers specific to each cell; cancer cells (orange), stromal cells (green), CD4+ T-cells (yellow), CD8+ T-cells (pink). A combination of the tissue segmentation and cell phenotyping enabled separate assessments of infiltrating immune cells in intratumoral and stromal regions. [file 13058_2022_1563_MOESM2_ESM.tif]

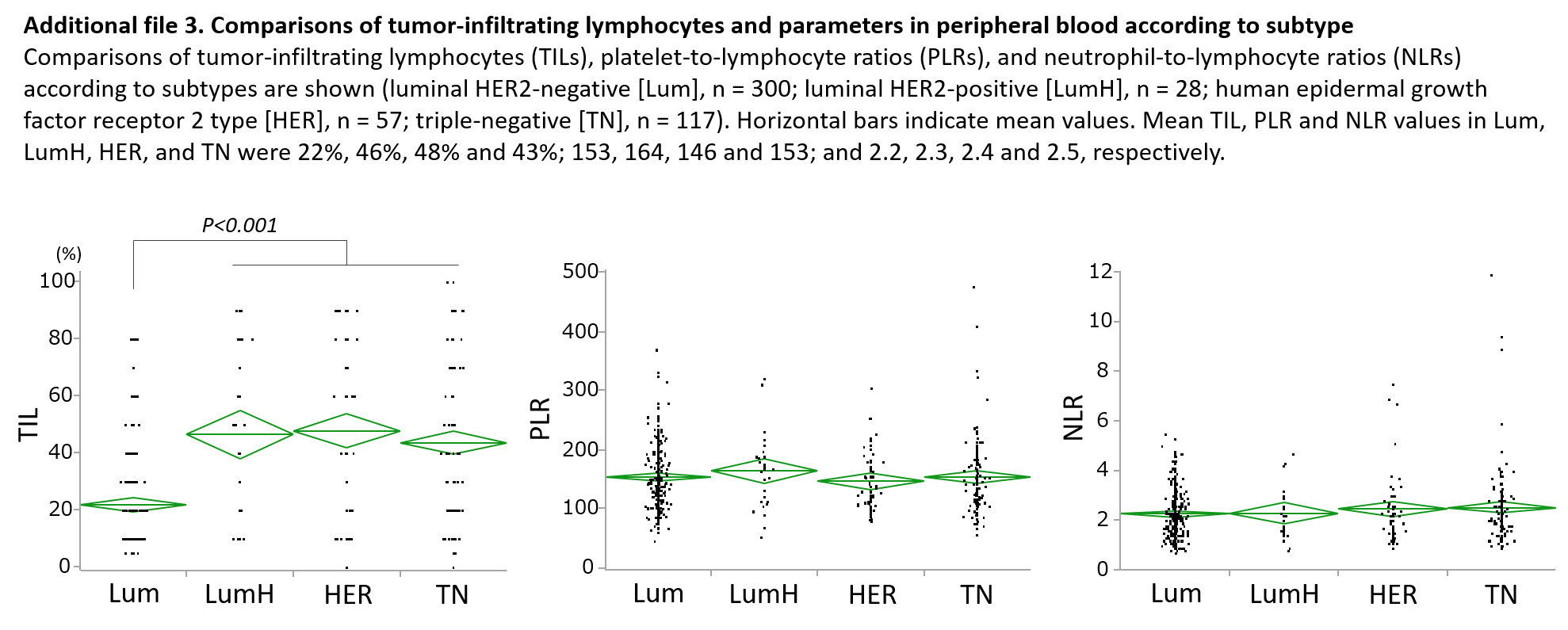

Supplement: Supplementary file 3 — Additional file3. Comparisons of tumor-infiltrating lymphocytes and parameters in peripheral blood according to subtype. Comparisons of tumor-infiltrating lymphocytes (TILs), platelet-to-lymphocyte ratios (PLRs), and neutrophil-to-lymphocyte ratios (NLRs) according to subtypes are shown (luminal HER2-negative [Lum], n = 300; luminal HER2-positive [LumH], n = 28; human epidermal growth factor receptor 2 type [HER], n = 57; triple-negative [TN], n = 117). Horizontal bars indicate mean values. Mean TIL, PLR and NLR values in Lum, LumH, HER, and TN were 22%, 46%, 48%, and 43%; 153, 164, 146, and 153; and 2.2, 2.3, 2.4, and 2.5, respectively. [file 13058_2022_1563_MOESM3_ESM.tif]

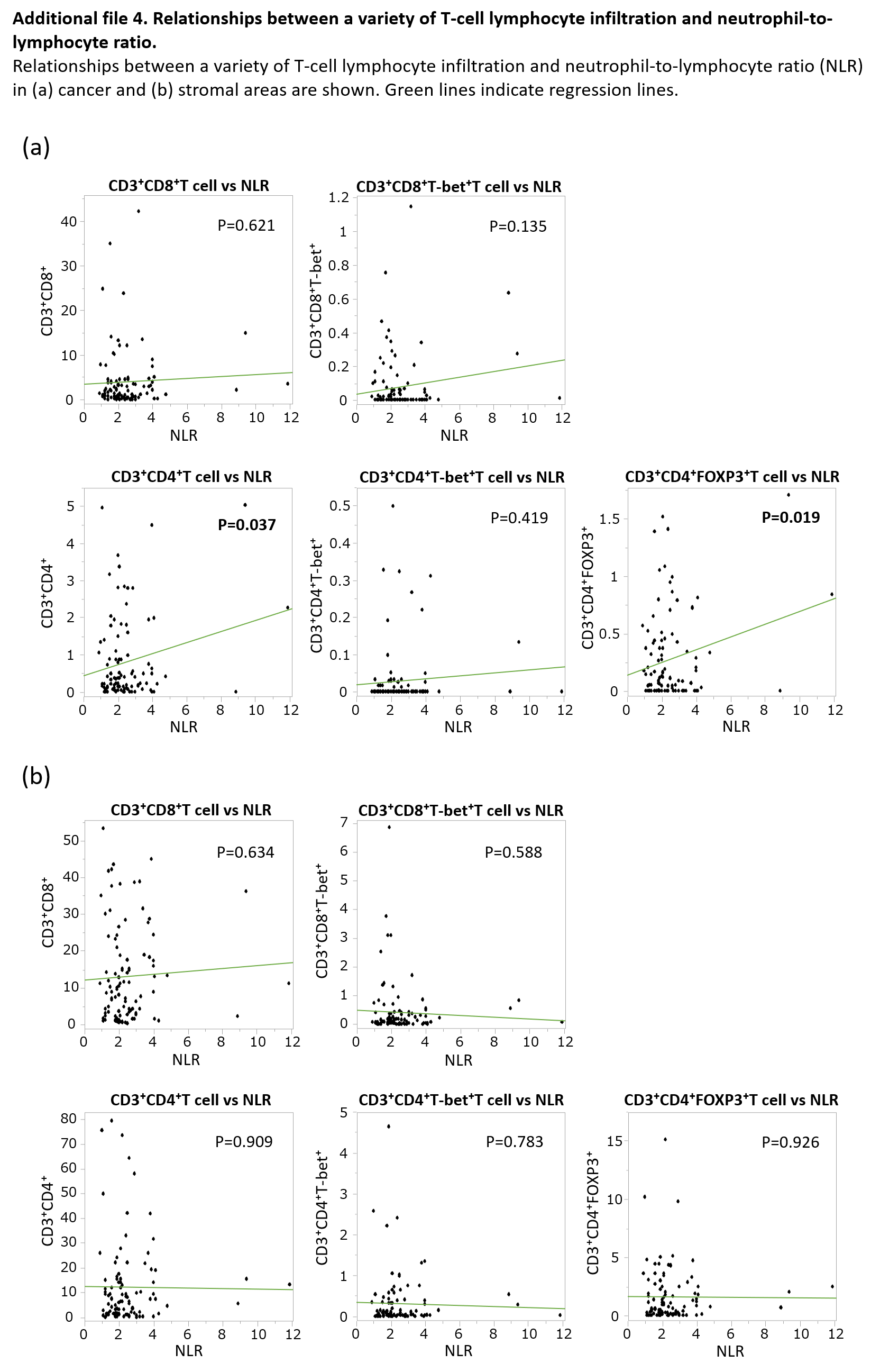

Supplement: Supplementary file 4 — Additional file4. Relationships between T-cell lymphocyte infiltration and neutrophil-to-lymphocyte ratio. Description of data: Relationships between a variety of T-cell lymphocyte infiltration and neutrophil-to-lymphocyte ratio (NLR) in (a) cancer and (b) stromal areas are shown. Green lines indicate regression lines. [file 13058_2022_1563_MOESM4_ESM.tif]

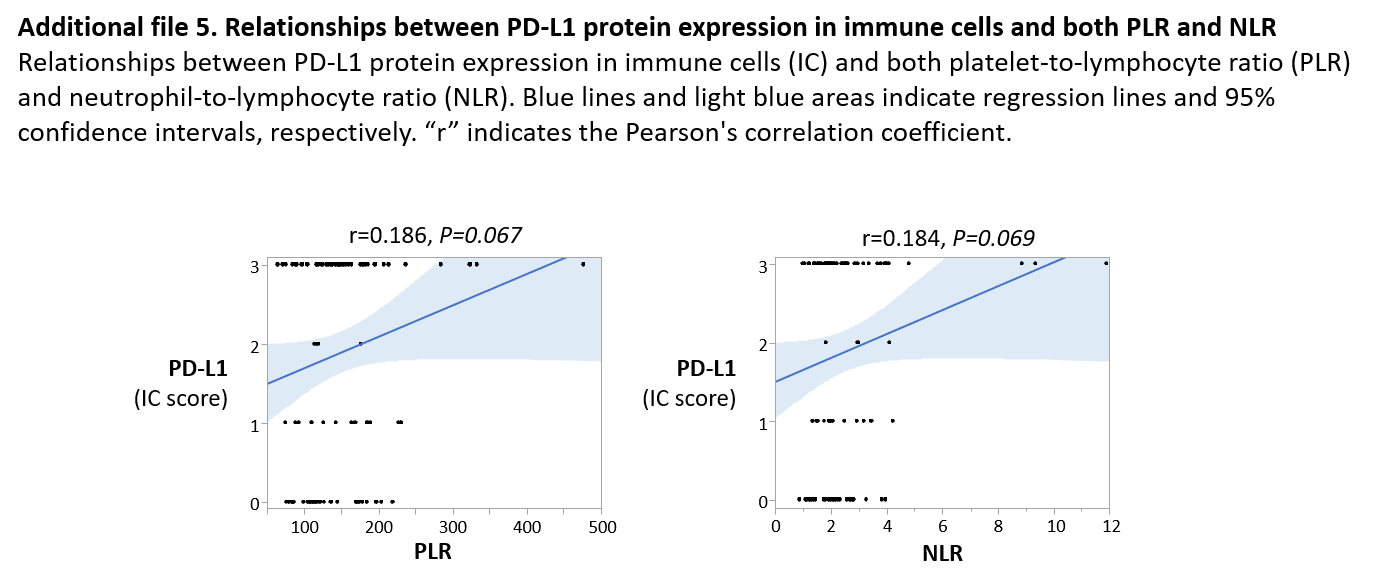

Supplement: Supplementary file 5 — Additional file5. Relationships between PD-L1 protein expression in immune cells and both PLR and NLR. Relationships between PD-L1 protein expression in immune cells (IC) and both platelet-to-lymphocyte ratio (PLR) and neutrophil-to-lymphocyte ratio (NLR). Blue lines and light blue areas indicate regression lines and 95% confidence intervals, respectively. “r” indicates the Pearson's correlation coefficient. [file 13058_2022_1563_MOESM5_ESM.tif]

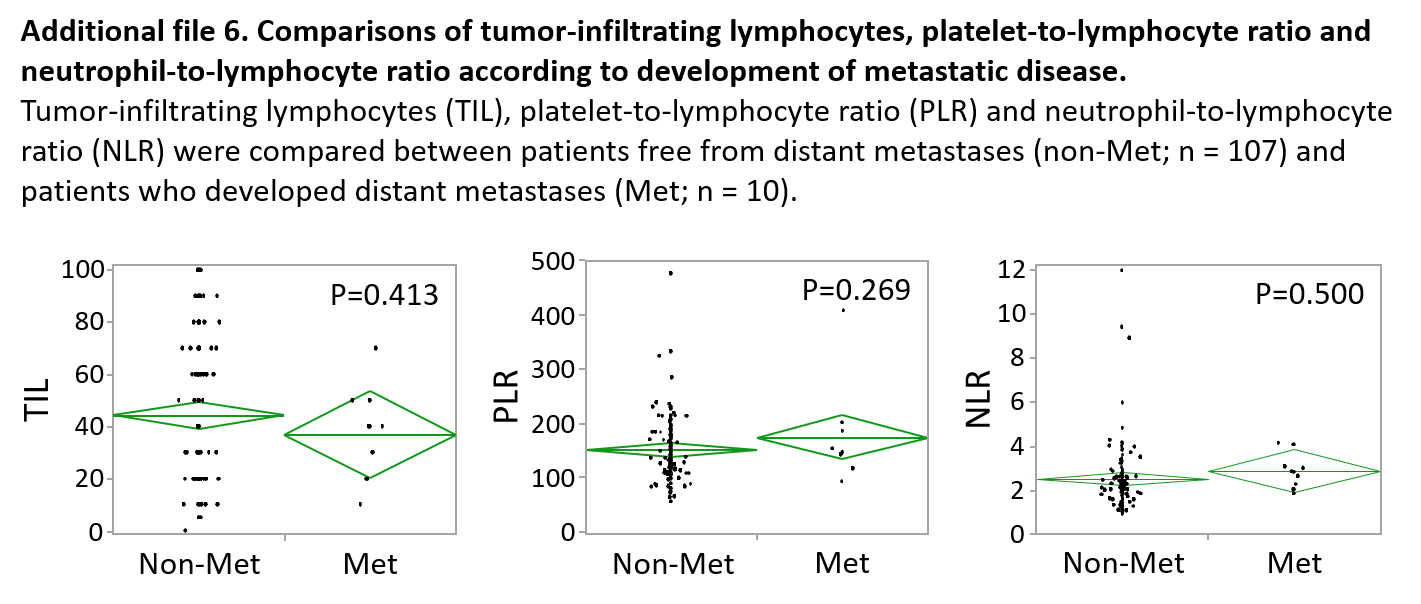

Supplement: Supplementary file 6 — Additional file6. Comparisons of tumor-infiltrating lymphocytes, platelet-to-lymphocyte ratio and neutrophil-to-lymphocyte ratio according to development of metastatic disease. Tumor-infiltrating lymphocytes (TIL), platelet-to-lymphocyte ratio (PLR) and neutrophil-to-lymphocyte ratio (NLR) were compared between patients free from distant metastases (non-Met; n = 107) and patients who developed distant metastases (Met; n = 10). [file 13058_2022_1563_MOESM6_ESM.tif]
